# Engineering HSV-1 for oncolytic therapy: From molecular entry mechanisms to retargeting strategies

**DOI:** 10.1016/j.gendis.2025.101797

**Published:** 2025-08-08

**Authors:** Yufang Zou, Juan Tao, Yingzheng Gao, Jixuan Wang, Pengfei Wang, Jingyuan Yan, Zuqing Nie, Dewei Jiang, Xinwei Huang

**Affiliations:** a Key Laboratory of the Second Affiliated Hospital of Kunming Medical College, Kunming, Yunnan 650101, China; b Key Laboratory of Animal Models and Human Disease Mechanisms of Yunnan Province, Kunming Institute of Zoology, Chinese Academy of Sciences, Kunming, Yunnan 650201, China; c Kunming College of Life Sciences, University of the Chinese Academy of Sciences, Kunming, Yunnan 650204, China

**Keywords:** Cell fusion, Glycoprotein genetic engineering, Herpes simplex virus type I, Oncolytic virotherapy, Systemic administration, Tropism-retargeting, Virus entry

## Abstract

Oncolytic viruses (OVs) represent a cutting-edge approach to cancer immunotherapy, characterized by their ability to selectively infect and eliminate tumor cells while sparing healthy tissues. Among the diverse OVs, type 1 herpes simplex virus (HSV-1) stands out due to its robust oncolytic activity, genetic malleability, broad cell tropism, and well-documented clinical safety. Advances in genetic engineering have further amplified the therapeutic efficacy of HSV-1 by enhancing tumor specificity, replication efficiency, and immunogenicity. Clinically significant HSV-1-based oncolytic viruses, such as T-VEC and G47Δ, have gained regulatory approvals for treating melanoma and malignant glioma, respectively, highlighting their transformative potential in cancer therapy. The attenuation strategies employed in most oncolytic HSV-1 (oHSV-1) strains, while ensuring safety, often reduce viral replication and cytotoxicity. To address this limitation, retargeting strategies focusing on HSV-1 glycoproteins (gD, gH/gL, and gB) have been developed. These modifications aim to abolish canonical receptor interactions and achieve tumor-specific targeting through ligand-receptor binding. Recent breakthroughs in understanding HSV entry mechanisms have enabled the creation of fully retargeted HSV vectors with enhanced specificity and efficacy. This review explores the molecular mechanisms underlying HSV glycoprotein-mediated cell entry, examines recent advances in receptor-retargeted oHSV-1 engineering, and discusses the challenges and future directions in the development of oncolytic HSV-based therapies.

## Introduction

Oncolytic viruses (OVs) have emerged as promising immunotherapies in cancer therapy. These viruses are genetically modified to selectively infect and destroy tumor cells without affecting healthy cells. The key mechanism underlying OVs is their ability to induce immunogenic cell death in cancer cells, triggering an immune response against the tumor. Additionally, genetic engineering techniques offer opportunities to enhance the anti-tumor potential of OVs. By utilizing gene editing, it is possible to enhance the selectivity of OVs for tumors, improve their replication capacity within tumor cells, reduce pathogenicity, and strengthen their ability to elicit an immune response.^1^

The commonly used OVs include type 1 herpes simplex virus (HSV-1), adenovirus, poxvirus, Newcastle disease virus, and reovirus. Each OV has distinct cellular entry mechanisms and exhibits varied responses to different types of tumor cells.^2^ Among these, HSV-1 shows promising clinical applications due to its potent oncolytic ability, broad range of infected cells, ease of genetic manipulation, induction of long-term cellular immune response, and the availability of drugs for controlling its proliferation.^2^ Like most viruses, oncolytic HSV (oHSV) replicates more effectively in cancer cells with defects in anti-viral signaling or viral nucleotide metabolism-related pathways.^3^^,^^4^ This strategy of tumor-conditional regulation of oncolytic virus replication was initially applied in the field of oHSV-1, achieving significant success. To date, several oHSV-1 strains, including HSV1716,^5^, ^6^, ^7^ NV1020,^8^ G207,^9^^,^^10^ G47Δ,^11^ and T-VEC,^12^ have advanced to clinical stages. Notably, T-VEC became the first oncolytic immunotherapy approved by both the U.S. Food and Drug Administration and the European Medicines Agency in 2015,^13^ and G47Δ was approved in Japan for treating malignant glioma in 2021.^14^

However, mutations have weakened toxicity and replication to varying extents. Retargeted viruses, in contrast, achieve cancer specificity through specific tropism for receptors expressed by cancer cells without requiring attenuation.^15^ Complete redirection of viruses remains challenging due to the inherent natural tropism of various viral glycoproteins for certain cell types, tissues, and organs. Moreover, the infection route, ability to establish productive infections in different types of cells, and the virus-host immune interactions significantly influence viral tropism.^16^ Non-enveloped viruses use one or more viral capsid proteins that interact with host cells during infection. In contrast, HSV encodes 12 different glycoproteins (B, C, D, E, H, I, K, and L) that contribute to its entry into hosts and spread between cells. The diverse array of glycoproteins encoded by HSV presents a significant challenge when attempting to retarget this virus.^16^

Currently, oHSV-1 detargeting and retargeting primarily involve genetic modifications of four essential glycoproteins: gD, gH/gL, and gB. In theory, it is necessary to disrupt or eliminate all the interactions between the virus and canonical entry receptors, replacing them with ligand-receptor interactions, to achieve complete retargeting of HSV-1. The selection of alternative receptors is limited to those that serve as tumor-specific markers and can be recognized by peptide ligands or single-chain antibodies (scFvs or specific single-chain variable fragments) without activating the receptor's normal physiological functions. In recent years, extensive research conducted in various laboratories has significantly enhanced our understanding of HSV entry mechanisms, ultimately enabling the development of fully retargeted HSV vectors.^16^ This review primarily focuses on the molecular mechanisms of viral glycoprotein-mediated cell entry in HSV infection, and highlights advances in receptor-retargeted oHSV developments that rely on the structural and functional properties of these glycoproteins. Ultimately, it outlines the opportunities and challenges in the field of oncolytic HSV development.

## HSV cell entry mechanism

**Figure 1:**
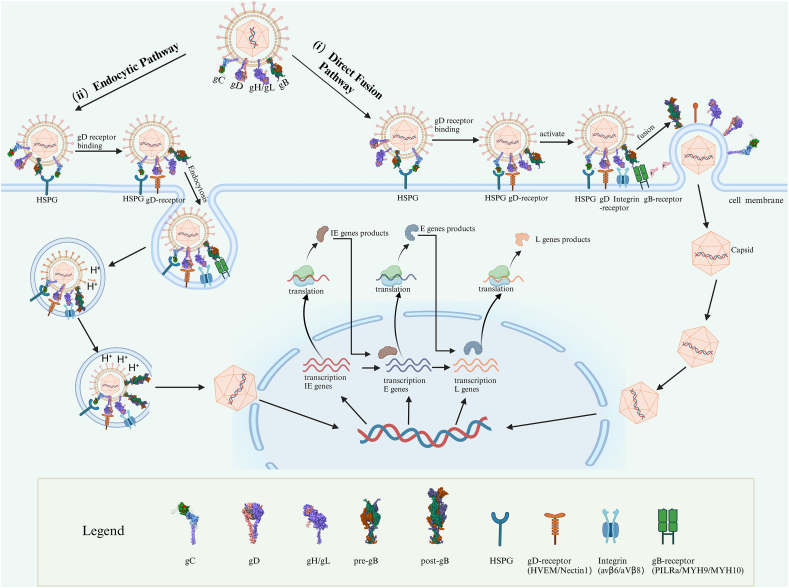
Models of HSV-1 entry into host cells. **(i)** Direct fusion pathway: HSV-1 initially attaches to the host cell surface via the binding of gC and/or gB to heparan sulfate proteoglycans (HSPGs). This attachment is followed by the binding of gD to its specific receptors, such as nectin-1. The receptor binding induces a conformational change in gD, transitioning it from a self-inhibited state to an active conformation that exposes its pre-fusion domain (PFD). This activation signal is transmitted to the gH/gL heterodimer. Concurrently, the gH/gL complex binds to integrin receptors on the host cell surface, causing the dissociation of gL from the complex and activating gH. Activated gH interacts with the cytoplasmic domain (CTD) of gB, triggering a conformational shift in gB from its pre-fusion to post-fusion state. This shift facilitates the fusion of the viral envelope with the host cell membrane, releasing the viral DNA into the host nucleus to initiate replication. **(ii)** Endocytic pathway: In some cell types, the gH/gL complex activates integrin receptors, promoting viral endocytosis through signaling via the C-terminal tail of the integrin β subunit. Within the acidic environment of the endosome, gB is activated, facilitating the fusion of the viral envelope with the endosomal membrane. This process releases the viral capsid and tegument proteins into the cytoplasm. The viral DNA is subsequently transported to the nucleus, where it initiates transcription and replication. The figure was created with BioRender.com.

HSV-1 viral particles consist of an icosahedral nuclear capsid housing a 152 kb double-stranded DNA genome. Surrounding the capsid is a tegument layer of viral and cellular proteins, enclosed by a lipid membrane derived from the host cell. This membrane contains 12 types of viral glycoproteins, five of which (gB, gC, gD, gH, and gL) are essential for HSV-1 entry.^16^, ^17^, ^18^, ^19^, ^20^ Viral entry mechanisms vary depending on the cell type. In some cases, the viral envelope fuses directly with the cytoplasmic membrane, as observed in Vero and human epithelial cells.^21^ Alternatively, HSV can fuse with host endocytic membranes post-internalization via pH-dependent or pH-independent pathways involving gD, gB, and gH/gL (Fig. 1).^22^^,^^23^ These variations likely arise from differences in receptor expression levels and affinities across cell types, determining the specific entry pathways used by HSV-1.

## Direct virion-cell fusion pathway

During HSV-1 or HSV-2 infection, a multiprotein complex comprising glycoproteins gB, gH/gL heterodimer, and gD, along with their cognate receptors, forms the “core fusion machinery” essential for membrane fusion.^24^^,^^25^ Among these, gB acts as the key fusogen, and both gB and gH/gL exhibit high conservation within the herpesvirus family.

The membrane fusion process occurs in three stages (Fig. 1). Initially, gB or gC binds to heparan sulfate on the host cell surface, promoting viral adhesion. While infection can occur in the absence of HS or gC, this interaction significantly enhances infectivity.^26^ Next, gD interacts with receptors such as herpesvirus entry mediator (HVEM), nectin-1, or 3-O-sulfated heparan sulfate proteoglycan (3-OS-HS), stabilizing the virus-cell connection and triggering fusion. Finally, gD-receptor binding induces conformational changes that activate gH/gL, which in turn activate gB to execute membrane fusion.

**Figure 2:**
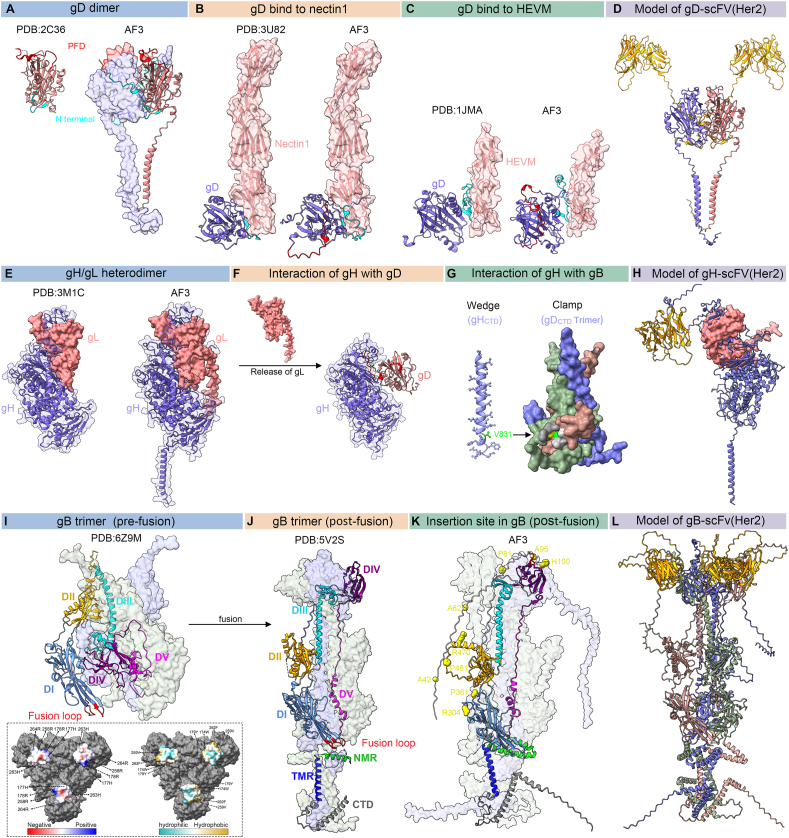
Structural basis of HSV-1 glycoproteins involved in viral entry. **(A)** Crystal structure of gD: The crystal structure of a monomer from the dimeric form of the gD ectodomain (PDB 2C36) is shown alongside AlphaFold3's prediction for the full-length gD dimer. The light purple surface highlights one monomer of the gD homodimer, allowing simultaneous visualization of the 3D surface and ribbon structure. The N-terminus (residues 6–38), responsible for nectin-1 or HVEM binding, is highlighted in cyan, while the pre-fusion domain (PFD, residues 260–285) involved in gD activation is highlighted in red. **(B, C)** gD-receptor interactions: The crystal structures of gD bound to its receptors nectin-1 (B) and HVEM (C) illustrate receptor-binding mechanisms. AlphaFold3's predictions extend these structures, showing the interaction between the N-terminus of gD and the receptor, along with the positional changes of the PFD domain not observed in the resolved structures. In both cases, the N-terminus of gD (cyan) forms a loop containing all receptor-contact residues. Receptor binding displaces the PFD (red), allowing interactions with gH. **(D)** Model of gD-Her2: A homodimeric model of gD engineered to target Her2. The anti-Her2 scFv (yellow) replaces the N-terminus of gD (residues 6–38). **(E)** Structure of gH/gL: The HSV-2 gH/gL heterodimer (PDB 3M1C) is shown from a side view alongside AlphaFold3's prediction for the full-length HSV-1 gH/gL heterodimer. **(F)** gH activation model: Upon binding to αVβ6 or αVβ8 integrins, gL dissociates, allowing gH to bind to receptor-activated gD. The ectodomain of gH interacts with the same surface previously occupied by gL. The PFD (red), along with an upstream 15-residue segment (green), forms a loop containing all contact residues (hydrogen bonds) for gH. These interactions between gH and gD were predicted using AlphaFold3. **(G)** Model of gH-gB interactions: A model depicting the interaction between the cytoplasmic domain of gH and the gB trimer (PDB 5V2S). The pocket on the gB_CTD_ surface is colored gray, with its base formed by residues T814 (yellow) and A851 (lime). According to the “clamp and wedge” model of gB activation, residue V831 (green) of gH wedges into this pocket, disrupting the inhibitory clamp and promoting the transition to post-fusion gB. **(H)** Model of gH/gL-Her2: A heterodimeric model of gH/gL engineered to target Her2. The anti-Her2 scFv (yellow) is inserted at residue 23 of gH. **(I, J)** Pre-fusion and post-fusion structures of gB: The crystal structures of the pre-fusion (I) and post-fusion (J) forms of the gB trimer are shown. The five domains of the gB ectodomain (DI to DV) are colored medium blue, orange, cyan, purple, and magenta, respectively. The fusion loops (red) in domain I form the trimer's legs, with the loops oriented toward the membrane. The box below shows the bottom of the pre-fusion gB trimer, and positively charged residues (177H, 178R, 258R, 263H, and 264R) and hydrophobic residues (174W, 179Y, 259V, and 262F) located in the fusion loops are highlighted. In the post-fusion gB structure, the membrane-proximal region (MPR, green), transmembrane region (TMR, blue), and cytoplasmic domain (CTD, dark gray) are visible. **(K)** Insertable sites in gB: Distribution of sites in gB that tolerate insertions without disrupting surface expression or fusion activity. AlphaFold3's predictions highlight residues capable of accommodating large insertions, such as fluorescent proteins, including A42, A62, P81, A95, H100, R304, P361, R470, and P481 (shown as space-filled residues). Most insertion sites are located in the unstructured N-terminal region of gB (white). **(L)** Model of gB-Her2: A post-fusion trimeric model of gB engineered to target Her2. The anti-Her2 scFv (yellow) is inserted at residue 42 of gB.

The gH/gL activation model proposed by Gianni et al suggests that receptor binding by gD and interaction of gH/gL with αvβ6 (or αvβ8)-integrins facilitate gL dissociation from the gH/gL complex, allowing gH to signal gB activation (Fig. 2E–G).^27^ This interaction also promotes endocytosis via the β-integrin C-tail. In this model, gL acts as an inhibitor, maintaining gH in its inactive form until receptor signals release it.

The subsequent activation of gB is explained by the “clamp and wedge” model, derived from structural and functional analyses of gB and gH cytotail mutants.^28^, ^29^, ^30^ According to this model, the cytoplasmic domain (CTD) of gB may act as a “clamp” to stabilize its prefusion state through interaction of the end of h2b and h3 with the membrane. Subsequently, upon interaction between the ectodomains of activated gH and gB, it causes transient interaction of gB CTD with gH cytotail. The gH cytotail then releases the gB's CTD clamp by wedging into the intertwined CTD (Fig. 2G). This release allowed gB to refold into its thermodynamically favored postfusion conformation, drawing its fusion loop onto the host's surface. The hydrophobic amino acids present in this fusion loop are believed to contribute to lipid fusion processes. In addition, cellular receptors for gB, such as non-muscle myosin heavy chain IIA (NMHC-IIA), non-muscle myosin heavy chain IIB (NMHC-IIB), paired immunoglobulin-like receptor A (PILRa), or myelin-associated glycoprotein (MAG), can play a significant role in the infection of HSV-1.^31^, ^32^, ^33^, ^34^ The NMHC-IIA, NMHC-IIB, and MAG not only facilitate virion attachment but also actively promote the fusion process between plasma membranes, which is evident from their ability to significantly enhance cell–cell fusion when gB, gD, gH, and gL were co-expressed.^31^^,^^32^^,^^34^

Following membrane fusion, HSV-1 releases its nucleocapsids and tegument proteins into the host cytoplasm. The nucleocapsids then interact with the nuclear membrane to deliver viral DNA into the nucleus, initiating transcription and replication of the viral genome.^35^

## Endocytic pathway of viral entry

HSV-1 can also enter host cells through a low pH-dependent endocytic pathway in various cell types. Enveloped virions can be readily observed in HeLa cells, primary human keratinocytes, and CHO–K1 cells transfected with gD receptor, and the entry of the virus into host cells can be impeded by lysosomotropic agents.^23^^,^^36^ Upon internalization, virions are transported to early endosomes where the reduced pH induces conformational changes in fusion proteins, enabling fusion between the viral envelope and vesicle membranes.^37^

Although the exact mechanisms triggering internalization remain unclear, the process resembles phagocytosis, involving actin cytoskeleton rearrangement mediated by RhoA signaling but independent of clathrin.^38^ Once internalized, the virus reaches early endosomes within the cell. The mildly acidic pH (pH 6.2–6.4) in these endosomes activates viral glycoproteins such as gB and gH/gL, without affecting the conformation or hydrophobicity of gD or its ability to bind to nectin-1 or HVEM receptors.^39^, ^40^, ^41^ Low pH induces changes in the antigenic conformation, hydrophobicity, and oligomeric structure of the prefusion form of gB, with only marginal effects on the postfusion form.^39^^,^^40^ Additionally, low pH can induce conformational changes in both gH and gL proteins, allowing physical interaction with gB.^41^ Subsequent fusion releases nucleocapsids and tegument proteins into the cytoplasm, completing viral entry.

## Glycoproteins for HSV-1 entry into cells and its receptors

### Glycoprotein C

The gC protein, encoded by the UL44 gene of HSV-1 and HSV-2, consists of 511 amino acids.^42^ It features multiple N-linked and O-linked oligosaccharides, along with a mucin-like region critical for interacting with glycosaminoglycans.^30^ HSV-1 entry into mammalian cells typically begins with the virus attaching to the cell surface, a process in which both gC and gB independently engage with heparan sulfate proteoglycans.^43^, ^44^, ^45^ While the gC–HS interaction enhances viral attachment, it is not essential for viral entry, as viruses lacking gC maintain infectivity in cultured cells.^44^^,^^45^ In addition to facilitating viral attachment, gC plays a critical role in immune evasion by binding to complement protein C3b, which disrupts the C3b-C5 interaction and inhibits complement activation, reducing viral clearance.^46^ Furthermore, recent studies reveal that the absence of gC enhances HSV-1 antibody neutralization, while its presence diminishes neutralization by a factor of 2–16.^47^

### Glycoprotein D and its receptors

After the initial interaction with cell surface heparan sulfate, the next stage of HSV entry involves the binding of gD to one of its specific receptors: HVEM, nectin-1, and 3-O-sulfated heparan sulfate (3-O-HS).^48^, ^49^, ^50^, ^51^, ^52^ This receptor diversity underpins the broad tropism of HSV-1, enabling infection across various cell types. The gD protein, encoded by the US6 gene of HSV, is a type I transmembrane protein comprising 369 amino acids. Its N-terminal extracellular region spans 316 amino acids.^53^ Crystallographic studies reveal that gD's external domain features an immunoglobulin-like core, flanked by N-terminal and C-terminal extensions.^54^ The N-terminal receptor-binding domain (RBD) specifically binds to host receptors, while the C-terminal pre-fusion domain (PFD) interacts with gH.^55^ Importantly, the PFD forms a self-inhibitory closed conformation with the RBD, preventing premature interactions with gH/gL or gB. Upon receptor binding, this conformation shifts, releasing the PFD to engage with gH/gL, a critical step in the fusion process (Fig. 2A–C).^54^^,^^56^ Surface plasmon resonance and epitope-mapping studies using anti-gD monoclonal antibodies have shown that gH/gL binds directly to gD at distinct sites, separating from the receptor-binding domain.^57^, ^58^, ^59^ These molecular insights into HSV entry mechanisms highlight potential targets for novel antiviral therapies. Additionally, gD facilitates the transmission of viral particles between cells and promotes the formation of polykaryocytes, underscoring its role in HSV pathogenesis.^60^ Immunization studies in mice using gD-expressing plasmids or gD-IL-2 DNA have demonstrated robust humoral and cellular immune responses, effectively mitigating corneal stromal inflammation.^61^

Nectin-1 and nectin-2 are type I transmembrane glycoproteins in the immunoglobulin superfamily, expressed across various human tissues and cell lines. They play a key role in cell–cell adhesion by interacting with nectins on adjacent cells.^51^ During HSV-1 entry, gD binds specifically to the variable region of nectin-1, facilitating viral recognition and attachment while disrupting nectin-mediated adhesion between neighboring cells.^62^^,^^63^ Interestingly, deletions in gD's N-terminal region (*e.g.*, residues 1–32) do not compromise binding efficiency to nectin-1.^55^ However, mutations at residues 215, 222, and 223, as well as variations in the N-terminal insertion length, can significantly affect binding efficiency.^64^^,^^65^ Similarly, the length of insertion at the N-terminus of gD also influences its binding properties with nectin-1. Notably, HSV-1 infection can still occur in neural and epithelial cells even in the absence of nectin-1, reflecting the virus's adaptive entry mechanisms.^48^

HVEM, the first identified receptor for HSV gD, is a type I transmembrane protein with four cysteine-rich domains (CRDs) in its extracellular region.^49^ CRD1 and CRD2 directly interact with gD, as revealed by crystallographic studies of the gD-HVEM complex.^66^ This interaction is essential for HSV-1 and HSV-2 infections, enabling viral attachment and membrane fusion.^25^ Belonging to the tumor necrosis factor (TNF) superfamily, HVEM is expressed in immune cells (*e.g.*, T cells, B cells, dendritic cells, macrophages, natural killer cells) and other cell types like neurons and fibroblasts, playing roles in inflammation and immune regulation.^67^^,^^68^ During HSV infection, gD-HVEM binding is critical for viral entry, facilitating fusion between the viral envelope and host cell membrane.

HSV-1 utilizes heparan sulfate as an initial attachment point on host cell surfaces. However, successful entry requires the modified form of HS, 3-O-HS, synthesized by 3-O-sulfotransferase.^69^ 3-O-HS acts as a distinct receptor for gD, complementing nectin-1 and HVEM in facilitating HSV-1 entry.^52^ Studies have shown that reduced 2-O-sulfation, mediated by decreased levels of 2-O-sulfotransferase, significantly impairs viral entry. Furthermore, these three gD receptors—nectin-1, HVEM, and 3-O-HS—appear to mediate membrane fusion via a shared mechanism.^70^

### HSV fusogen gB and its receptors

The glycoprotein B (gB) of HSV-1 is recognized as the fusion protein, belonging to the conserved class III fusion glycoproteins.^71^ The ectodomain of gB shares structural similarities with the post-fusion structures of vesicular stomatitis virus (VSV) G protein and baculovirus gp64, despite sequence variations.^71^, ^72^, ^73^ Encoded by the UL27 gene, gB exists in two forms: pre-fusion and post-fusion. While the post-fusion extracellular domain has been extensively studied through crystallography, the structure of the pre-fusion form has been resolved recently by introducing an H516P mutation to functionally lock the gB prefusion conformation.^74^ Crystallographic studies reveal that the post-fusion form of gB forms a rod-like structure with an α-helical trimer core. As a type I transmembrane protein, gB comprises an extracellular region (696 amino acids), a transmembrane region (69 amino acids), and an intracellular region (109 amino acids). Each subunit of the trimer consists of five distinct domains (I–V) and two connecting regions, forming a hairpin-like structure.^75^^,^^76^ Domain I contains hydrophobic fusion loops essential for membrane fusion; domain II facilitates interactions with gH/gL; domain III is composed of alpha helices, forming the trimeric helix–helix core. Domain IV is a crown-shaped structure implicated in receptor binding; antibody binding to this domain inhibits gB-receptor interactions.^77^, ^78^, ^79^ Domain V is an arm domain providing structural support through elongated subunit connections.^77^ Flexible linker regions enable conformational changes critical for gB-mediated fusion.^80^^,^^81^ While the N-terminal region (amino acids 31–102) lacks a defined role, its flexibility suggests potential, yet uncharacterized, functional significance (Fig. 2I, J).^24^

PILRα, a 303-amino-acid receptor, specifically binds to gB through its Ig-like V-type domain (amino acids 31–150), which is critical for membrane fusion activity. Substitution of tryptophan at position 139 disrupts gB binding and eliminates fusion activity.^82^ While predominantly expressed in immune cells (*e.g.*, monocytes, macrophages, dendritic cells), PILRα is also found in the nervous system, highlighting its potential role in HSV-1 infections across tissues. However, PILRα expression levels in most tissues, including peripheral blood mononuclear cells, are low, suggesting the involvement of additional receptors in mediating HSV-1 entry.^74^

Non-muscle myosin II (NM-II) is composed of heavy chains (NMHC), regulatory light chains, and essential light chains, and it regulates key cellular processes, including adhesion and migration.^83^^,^^84^ NMHC-IIA and NMHC-IIB are highly conserved isoforms, sharing 80% identity in amino acid sequences.^85^ NMHC-IIA is identified as a gB receptor, and its overexpression enhances susceptibility to HSV-1, while anti-NMHC-IIA antibodies inhibit infection in NMHC-IIA-expressing cells. The widespread expression of NMHC-IIA in various human tissues and cell types further supports its role as a functional gB receptor, facilitating broad-spectrum infectivity of HSV-1 both *in vitro* and *in vivo*.^31^^,^^46^ Whereas, NMHC-IIB is expressed predominantly in neuronal tissues and implicated in neural infections. Overexpression enhances susceptibility to HSV-1, while knockdown reduces viral infection and envelope glycoprotein-mediated cell fusion in COS-1 cells.^32^^,^^86^

MAG, a member of the paired receptor family like PILRα, is primarily expressed in neural tissues. HSV-1 gB binds specifically to MAG, facilitating viral entry into glial cells.^87^ Although MAG is not expressed in epithelial cells and is thus not a primary receptor for HSV-1, it may be exploited during acute infections to mediate neurological diseases.^77^

### Glycoprotein gH/gL and its receptors

The gH/gL complex plays a crucial role in HSV infection by mediating viral fusion with host cell membranes. Despite extensive research, the precise mechanisms of this fusion process remain unclear. It is known that gH/gL interacts with other viral glycoproteins, such as gB and gD, to regulate the fusion reaction. Studies on HSV-2 using neutralizing antibodies indicate that the formation of a gB-gH-gL complex is a prerequisite for membrane fusion.^88^ Similarly, investigations employing a quantitative split-luciferase approach demonstrate that HSV-1 entry glycoproteins (gD, gB, gH, and gL) form complexes prior to and during membrane fusion. The interactions of these pairwise glycoproteins remain stable throughout the fusion process and are independent of the presence of cellular receptors.^89^

The gH protein, encoded by the UL22 gene, consists of 838 amino acids and functions as a type I transmembrane glycoprotein with a transmembrane domain and cytoplasmic tail.^90^ In contrast, gL, encoded by the UL1 gene, is a 224 amino acid-long protein lacking a transmembrane domain. It binds non-covalently to the N-terminus of gH, forming the heterodimeric gH/gL complex, which associates with the host cell plasma membrane (Fig. 2E).^91^^,^^92^ Furthermore, gH/gL binding to αvβ3 integrins or Toll-like receptor 2 (TLR2) has been shown to trigger NF-κB activation, eliciting innate immune responses.^93^^,^^94^

Highly conserved across herpesviruses, the gH/gL complex plays a crucial role in facilitating viral entry through endocytic pathways by interacting with host cell proteins, particularly integrins such as αvβ3, αvβ6, and αvβ8.^95^^,^^96^ Before receptor binding, gL maintains gH in an inactive state. Upon activation signals from receptor-bound gD and integrins, gL dissociates, activating gH. The activated gH binds to receptor-activated gD, and in turn, triggers gB activation through interactions mediated by their cytoplasmic domains (Fig. 2F, G). Mutations in the cytoplasmic domain of gH impair its fusion activity, highlighting its critical role in regulating gB-mediated membrane fusion.^97^ Recent studies suggest that the gB cytoplasmic tail contains a specific pocket that binds to gH residues (*e.g.*, V831 and S830). This interaction is believed to activate gB, facilitating membrane fusion (Fig. 2G).^98^^,^^99^

Among the αv integrin family (αvβ3, αvβ5, αvβ6, and αvβ8), αvβ6 and αvβ8 integrins exhibit high-affinity binding to gH, albeit at different locations.^96^ The interaction between αvβ6 and gH depends on the presence of the Arg-Gly-Asp (RGD) motif within the integrin-binding domain of gH, while αvβ8 binding is RGD-independent. αvβ6 integrins are predominantly expressed in epithelial cells, whereas αvβ8 integrins are highly expressed in the central nervous system, aligning with the primary targets of HSV infection.^100^^,^^101^ Although αvβ3 integrins bind to gH/gL with low affinity and do not induce fusion, they may assist HSV entry via an acidic endosomal pathway.^102^^,^^103^

## Strategy for tropism retargeted oHSV-1

### Conditionally replicating oHSVs

The tumor-specific oHSV-1 is engineered through genetic modification, primarily involving the mutation or deletion of viral replication regulatory genes. These strategies restrict viral replication in non-dividing cells while enabling replication in tumor cells, resulting in targeted tumor destruction. Currently, most strategies for modifying oHSV focus on viral nucleotide metabolism-related genes (UL39 and UL23), as well as the neurovirulence gene ICP34.5 (encoding γ34.5), which endows oHSV with tumor-specific cytotoxicity through different mechanisms.^3^^,^^4^ For instance, the single mutant oHSV hrR3 strain lacks the UL39 gene (encoding ICP6, a large subunit of ribonucleotide reductase),^104^ while the dlsptk strain lacks UL23 (encoding TK, thymidine kinase).^105^ The absence of these nucleotide metabolism-related genes allows the virus to efficiently replicate only in rapidly growing tumor cells, thereby possessing specific tumor lytic activity. In contrast, most HSV-1 oncolytic viruses carry Δγ34.5 mutations as a key factor ensuring tumor-specific replication and safety. As a critical neurovirulence gene, γ34.5 regulates various aspects, including protein translation systems, cellular autophagy, innate antiviral immunity, and viral particle release.^106^ Studies have shown that γ34.5 targets RIG-I,^107^ downstream adaptor molecule TBK-1,^108^ and STING,^109^ which inhibits immune activation and interferon production. Additionally, γ34.5 can suppress PKR activation by regulating eIFα, thus promoting viral protein synthesis.^110^ In tumor cells, the commonly existing MAP/ERK sustained activation signal inhibits PKR activity, which partially compensates for γ34.5-mediated resistance against the IFN anti-viral pathway, enabling γ34.5-deficient strains to replicate to some extent.^111^

To reduce the possibility of recombination and strain reversion within the body, some oHSV strains employ a multi-gene knockout strategy to further enhance their safety. For instance, recombinant oHSV strains R3616^112^ and 1716^113^ have two copies of γ34.5 deleted (which are present in two inverted repeat sequences flanking the UL region). In the G207 strain, both copies of the γ34.5 gene are excised while simultaneously inactivating the UL39 gene through LacZ insertion.^114^ Additionally, the G47Δ strain further knocks out US12 (encoding ICP47) to enhance MHCI-dependent antigen presentation by the virus.^115^

Transcriptional reprogramming is another potential strategy to restrict the replication of oncolytic viruses in tumor cells. This approach involves placing a crucial viral gene under the control of a promoter that is only active in cancer cells. For instance, by utilizing the Musashi1 promoter, the expression of the γ34.5 gene can be limited specifically to malignant glioma cells.^116^ Similar techniques have been employed for prostate tumors, where regulation of essential ICP27 gene transcription has been utilized to achieve effective virus replication in prostate cells.^117^ Additionally, liver cancer-specific promoters^118^ and hypoxia-inducible factor (HIF) responsive promoters^119^ regulating essential ICP4 gene for virus replication have also been investigated. To further minimize off-target infections, microRNA target sequences are incorporated into these strategies. Glorioso and Grandi successfully inserted an miR-124 target sequence into the ICP4 gene, which is expressed in neurons but absent in glioblastoma cells. This targeted modification protects healthy tissues from viral replication.^120^

### oHSV retargeting by glycoprotein engineering

**Figure 3:**
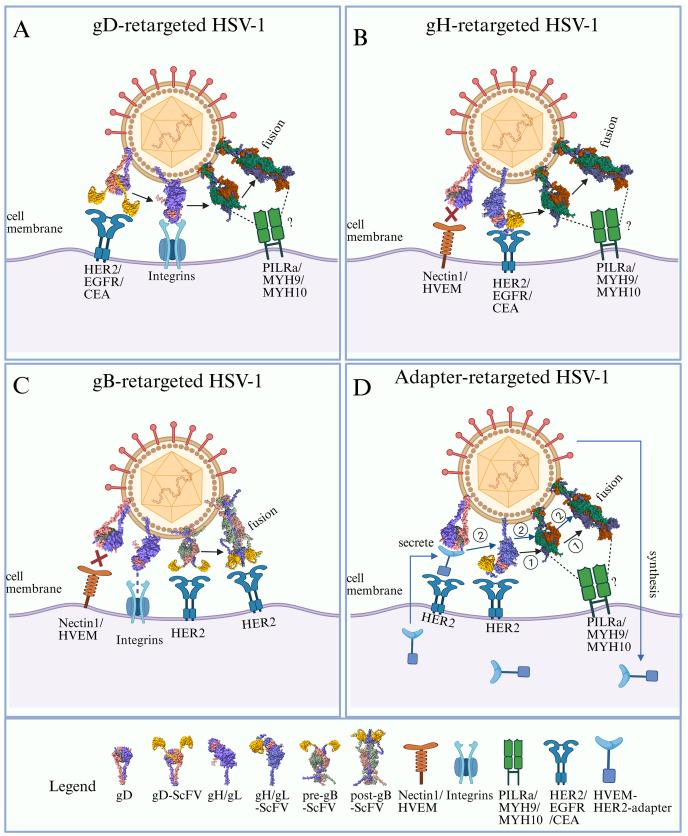
Strategies for retargeting oncolytic HSV-1. **(A)** gD modification for retargeting: Retargeting is achieved by inserting specific single-chain variable fragments (scFvs) into gD genes with deletions at amino acid (AA) positions 6–38. These modifications block the interaction between gD and its natural receptors, redirecting the virus to target cells expressing the corresponding receptor for the inserted scFv. Binding of the scFv-gD chimera to the new receptor activates gD, which transmits the activation signal to gH/gL and subsequently to gB, triggering viral fusion with the host cell membrane. The precise role of gB's receptor in facilitating entry, through either pre- or post-fusion forms, remains unclear. **(B)** gH modification for retargeting: Retargeting is accomplished by inserting specific scFvs between residues 23 and 24 of gH, enabling the virus to target cells expressing specific receptors. Concurrently, deletion of gD residues 6–38 prevents the interaction with natural gD receptors. In the absence of gD receptor activation, the interaction of the chimeric scFv-gH with specific receptors and integrins activates gH/gL, which subsequently activates gB, leading to fusion of the viral and host cell membranes. **(C)** gB modification for retargeting: Redirection consists of the insertion of anti-HER2 scFv at amino acid 42 (AA) at gB while removing gD residues 6–38 to prevent off-target interactions with gD native receptors. The interaction of the chimeric gB-HER2 with HER2 receptors directly activates gB, triggering membrane fusion and repositioning the virus's tropism to HER2-positive cells. **(D)** Retargeting via soluble adaptor molecules: The soluble adapter molecule consists of two functional domains: one end binds to HVEM in HSV gD, and the other end binds to a specific target receptor HER2 on host cells. The retargeting was achieved by inserting an expression cassette for the adapter HVEM-scFvHER2 in the UL3-UL4 intergenic region, while an open reading frame for anti-HER2scFv was inserted into residue 29 of gH. When adaptor proteins are properly synthesized and secreted, linkers bind to gD, and the target receptor HER2 activates gH/gL, then relays the activation signal to gB, and initiates fusion of the viral membrane with the host cell membrane.

In most cases, the cancer specificity of recombinant oHSV-1 is typically achieved through multiple mutations, often at the cost of attenuation, resulting in significantly lower cytotoxicity compared with its wild-type counterpart. To address these limitations and enhance oHSV-1 efficacy, an alternative strategy involves redirecting viral tropism to cancer-specific receptors while detargeting natural receptors. This strategy enhances cancer specificity without requiring gene deletions, allowing the virus to retain oncolytic activity comparable to wild-type HSV.^121^ Glycoproteins gD, gH, and gB have emerged as primary targets for these redirection strategies, which largely exploit membrane fusion mechanisms to mediate viral entry (Fig. 3). Successful retargeting has been demonstrated against a range of tumor-associated receptors, including IL13Rα2, uPAR, GFRα1, HER2, EGFR, PSMA, and EGFRvIII, enabling selective infection of various cancer cell types (Table 1).

**Table 1:** Retargeting strategies utilizing glycoprotein modification.

oHSV-1 name	Retargeting ligand(s) @ viral glycoprotein			Additional modifications		Target heterologous receptor	Parental strain	Reference
	gD	gB	gH/gL	Modification	Site			
R5111	IL-13 @gDaa24	HS-	WT			IL-13Ra2	HSV-1(F)	122
R5141	IL-13 @gDΔ1–32, gDV34S	HS-	WT			IL-13Ra2	HSV-1(F)	123
R5181	UPA @gDaa24	HS-	WT			UPAR	HSV-1(F)	124
R-LM11	scFv-HER2 @gDaa24	WT	WT			HER2	HSV-1(F)BAC	126
R-LM113	scFv-HER2 @gDΔ6-38	WT	WT	EGFP	UL3-UL4	HER2	HSV-1(F)BAC	127
R-LM249	scFv-HER2 @gDΔ61-218	WT	WT	EGFP	UL3-UL4	HER2	HSV-1(F)BAC	128
KNTCgD:GDNF	GDNF @gDΔ2–24, gDΔ38/Y38	gB:NT	WT	mcherry	UL3-UL4	GFRa1	HSV-1(F)BAC	119
R-611	scFv-EGFR @gDΔ6-38	WT	WT	EGFP	UL3-UL4	EGFR	HSV-1(F)BAC	130
R-593	scFv-PSMA @gDΔ6-38	WT	WT	EGFP	UL3-UL4	PSMA	HSV-1(F)BAC	130
R-613	scFV-EGFRVIII @gDΔaa6-38	WT	WT	EGFP	UL3-UL4	EGFRVIII	HSV-1(F)BAC	130
KG4:T124:ΔgD	ScEGFR/VHH/ZEGFR @gDΔaa2-24, @ gDΔaa6-24, @gDΔaa7-24, @gDaa38	gB:NT	WT	miR-124, T2A-GFP	ICP4, gC	EGFR	HSV-1(F)BAC	131
KNE	scFv-EGFR @ gDΔaa2-24, gDY38C	gB:NT	WT			EGFR, EGFRVIII	HSV-1(KOS)BAC	133
KNC	scFv-CEA @gDΔaa2-24, gDY38C	gB:NT	WT			CEA	HSV-1(KOS)BAC	133
R-87, R-89, R-97, R-99, R-99-2	scFv-HER2, GCN4 pep @gDaa24, @gDΔaa35-39, @gDΔaa214-223,	WT	WT	EGFP	UL3-UL4	GCN4, HER2	HSV1(F)BAC + EGFP	140
R-903	WT	scFv-HER2 @gBaa43-44	WT	EGFP	UL3-UL4	HER2	HSV-1(F)BAC	137
R-909	gDΔaa6-38	scFv-HER2 @gBaa43-44	WT	EGFP	UL3-UL4	HER2	HSV-1(F)BAC	137
R313, R-315, R317, R-319, R-321	gDΔ6–38, gDΔ30,38	GCN4 peptide and scFv-HER2 @gBΔaa43-44, @gBΔaa81-82, @gBΔaa76-77, @gBΔaa95-96	WT	EGFP	UL3-UL4	GCN4, HER2	HSV-1(F)BAC	138
R-VG803	WT	WT	scFv-HER2 @ gHΔaa23-24	mCherry	UL37-UL38	HER2	HSV-1(F)BAC	141
R-VG809	gDΔaa6-38	WT	scFv-HER2 @ gHΔaa23-24	mCherry	UL37-UL38	HER2	HSV-1(F)BAC	141
R-213	gDΔaa6-38	WT	scFv-HER2, GCN4 pep@gHΔaa23-24	EGFP	UL3-UL4	HER2, GCN4	HSV-1(F)BAC	139
scFv-EGFR: Nectin1 adapter	WT	WT	WT			scFv-EGFR		144
scFv-CEA: HVEM/Nectin-1 adapter	WT	WT	WT			scFv-CEA		145
scFv-EpCAM/HER2: HVEM adapter	R222N/F223I	WT	scFv-EpCAM/HER2 @gHΔaa29	GFP	U26/27	HVEM, ScFv-EpCAM	HSV-1(KOS)BAC	147

#### HSV retargeting via gD modification

Currently, it is well-established that HSV entry involves a multi-step process, including attachment mediated by glycoprotein gC and four essential glycoproteins: gD, gH/gL, and gB. Among these, gD is species-specific and serves as the major determinant of HSV-1 tropism.

The first retargeted oHSV-1, R5111, was developed by Zhou et al to target IL-13Rα2, a receptor overexpressed on malignant glioblastomas and high-grade astrocytomas.^122^ Modifications included deleting the HS-binding site in gB (Δaa 68–76), replacing the gC N-terminal domain (aa 1–136) with IL-13, and inserting IL-13 into the N-terminal of gD (aa 24). This recombinant virus retained the ability to bind to natural receptors HVEM and nectin-1 while targeting IL-13Rα2. To fully retarget R5111, R5141 was developed by deleting residues 1–32 of gD (the HVEM-binding region) and introducing a V34S mutation to disrupt nectin-1 binding. As a result, R5141 exclusively targeted IL-13Rα2-positive cells.^123^ R5181 was similarly engineered to target the urokinase plasminogen activator receptor (uPAR) in glioblastomas by inserting uPA between gD residues 24 and 25.^124^ For breast cancer, KNTc-gD:GDNF retargeted GFRα1 by replacing the gD signal peptide and HVEM-binding domain with pre-pro-GDNF and deleting aa 38 to prevent nectin-1 binding.^125^ This virus selectively infected GFRα1-positive breast cancer cells, leading to tumor regression *in vivo*.

HER2, a marker overexpressed in aggressive mammary and ovarian tumors, has also been targeted using gD modifications. Recombinant oHSV-1 strains R-LM11 and R-LM11L, which incorporated a single-chain antibody (scFv) targeting HER2 at gD residue 24, exhibited specificity for HER2-positive cells.^126^ Further modifications in R-LM113 or R-LM249 replaced gD residues 6–38 or the Ig-folded core (aa 61–218) with anti-HER2 scFv, achieving complete detargeting from natural receptors.^127^^,^^128^ Both strains showed efficacy in preclinical models of HER2-positive ovarian cancer and breast cancer, including peritoneal and brain metastases.^128^ Notably, intraperitoneal injection of R-LM249 showed therapeutic effects on peritoneal and brain metastases in ovarian and breast cancer models.^128^^,^^129^ These studies suggest that gD can tolerate fusion to large heterologous proteins without loss of ability to mediate virus entry. Furthermore, this paradigm can be extended to various types of tumors by substituting different scFvs.

Recent advances have established a highly versatile platform for oHSV retargeting by inserting scFv at the gD N-terminal (Δaa 6–38).^130^ This platform enabled the development of oHSVs targeting EGFR, PSMA, and glioblastoma-specific EGFRvIII. While scFv confers high specificity, research indicates that smaller ligands (*e.g.*, affibody molecules or VHH antibodies) improve virus production and packaging efficiency while retaining tumor-specific targeting.^131^ This clearly emphasizes the criticality of maintaining gD's membrane pro-fusion ability in preserving the high infectivity of the recombinant virus during gD retargeting modifications.

In addition, hyperfusogenic mutations in gB significantly enhance the infectivity and dissemination of gD-retargeted viruses. Joseph C. Glorioso et al have identified a mutant clone (D285N/A549T) of gB that compensates for gD-dependent virus entry through continuous passage screening using gD mutant strains in a HevA mutant cell line.^132^ In the gD retargeted virus (anti-EGFR or anti-CEA scFv insertion into aa 2–24), the introduction of D285N/A549T in gB increased the efficiency of retargeted virus entry, which achieved infectivity comparable to that of the wild-type virus via the standard entry receptors.^133^ Similarly, Laura Menotti also verified that this gB hyperfusogenic mutation could increase the ability of an HER2-retargeted recombinant to spread among tumor cells.^130^ Remarkably, the entry of retargeted virus accelerated by hyperfusogenic gB still relies on the interaction of gD or retargeted gD with its receptors. Hence, the hyperfusogenic mutation of gB may only lower the threshold for activation of virus fusion machinery, and it cannot independently mediate viral entry into cells.^55^^,^^132^

#### HSV retargeting via gB modification

HSV gB is a highly conserved class III fusion glycoprotein essential for HSV infection. X-ray crystallography studies have revealed that the post-fusion ectodomain of gB forms a trimeric structure composed of three protomers, creating a spike-like configuration.^71^ By mapping neutralizing monoclonal antibody epitopes onto the gB structure, functional regions (FR) of gB have been categorized as follows: FR1 (domains I and V), FR2 (domain II), FR3 (domain IV), and FR4 (the unresolved N-terminal region).^134^

Given its critical role in membrane fusion, modifications to gB must carefully preserve its fusogenic properties. Initial studies introduced the GFP coding sequence at the NotI site within the disordered N-terminal region of gB. This modification preserved gB's functionality and viral infectivity.^135^ Similarly, random linker-insertion mutagenesis studies (5-amino-acid insertion) revealed that the N-terminus and cytoplasmic tail of gB tolerate small insertions without affecting cell surface expression or fusion activity. While insertions in FR2 also tolerated modifications, they somewhat affected fusion and viral entry efficiency.^81^ Another study using 2-amino-acid insertions identified additional tolerant sites, including positions in the N-terminus, FR1, and FR2.^136^ Comprehensive mapping of fluorescent protein insertions across the ectodomain and N-terminus found eight functional insertion sites in FR1, FR2, and FR4, all maintaining membrane fusion ability.^127^ Collectively, these studies demonstrate that disordered regions in the gB N-terminus and FR2 domain are suitable for incorporating exogenous receptor-targeting ligands while preserving gB's fusogenic activity.

The first tumor-targeted application of gB modification was carried out by Gabriella Campadelli-Fiume's group, who constructed the R-909 strain. They inserted anti-HER2 scFv at aa 43 in gB and further deleted gD residues 6–38 to detarget the virus from natural gD receptors.^137^ This chimeric gB-HER2 construct redirected viral tropism to HER2-positive cancer cells. Interestingly, gB-HER2-induced membrane fusion did not require gD or gH/gL activation. Instead, entry relied partially on endocytic pathways, as indicated by the BFLA inhibition model. Furthermore, soluble HER2 protein activated viral glycoproteins, facilitating entry into cells. Interestingly, all Her2-targeted recombinant viruses, such as gD-Her2 (R-LM113), gH-Her2 (R-809), and gB-Her2 (R-909), can be activated by soluble Her2 protein and enter cells.^137^ This finding suggests that viral entry depends less on anchoring particles to cell surfaces and more on receptor-induced conformational changes in glycoproteins.

To address challenges in cultivating gD-targeted viruses in cell lines lacking tumor-specific receptors, Biljana Petrovic et al introduced a 20-aa GCN4 peptide into gB at several positions (aa 43–44, 76–77, 81–82, and 95–96) on the background of the gD-HER2 virus (R-LM113). The resulting strains (R-313, R-315, R-317, and R-319) successfully replicated and spread in Vero-GCN4R cells, a cell line expressing the complementary GCN4 receptor.^138^ Additionally, GCN4 peptides have been inserted into modified gH or gD proteins, enabling retargeted oHSVs to amplify efficiently in complementary cell lines.^139^^,^^140^ This strategy improves clinical-grade virus production, supporting the scalability of retargeted oHSV for therapeutic applications.

#### HSV retargeting via gH modification

The gH/gL heterodimer is a conserved feature across all herpesviruses. In HSV, the gH/gL complex interacts with two interchangeable receptors, αvβ6 and αvβ8 integrins, which promote virus internalization and facilitate gL displacement from the gH/gL complex, a likely step in gH activation.^27^^,^^96^ This interaction underscores gH's potential as a target for modifying oHSV-1 tropism by introducing exogenous ligands, provided the modifications minimally disrupt its interactions with gL and gB.

Gabriella Campadelli-Fiume's group pioneered gH-based retargeting by developing R-VG809, an oHSV strain engineered to target HER2-positive cells. This strain was constructed by inserting HER2-scFv at gH amino acid 23 and deleting residues 6–38 in gD, which include nectin-1 and HVEM binding sites. R-VG809 demonstrated specific replication and efficient killing of HER2-positive cells without requiring gD activation by its natural receptors.^141^ Compared with gD-based retargeting strategies, gH offers several advantages. In gD-based approaches, inserting large exogenous ligands can significantly alter gD's structural and functional properties, compromising the activation of gH/gL and gB. These limitations often necessitate additional modifications, such as introducing hyperfusogenic mutations in gB to compensate for deficiencies in chimeric gD. In contrast, gH appears more amenable to modification, with fewer functional disruptions when integrating exogenous ligands. This flexibility not only expands the design toolkit for oHSV-1 retargeting but also provides valuable insights into the roles and mechanisms of viral glycoproteins in mediating virus entry.

#### HSV retargeting by soluble adapters

Research on adenovirus retargeting has demonstrated the utility of bispecific soluble proteins (adapters). These adapters consist of a viral receptor-binding domain fused to ligands or scFvs targeting specific membrane proteins, which can redirect viral tropism while inhibiting interaction with natural receptors.^142^^,^^143^ For HSV-1, given the critical role of gD in mediating viral membrane fusion, early adapter designs included gD-binding domains (derived from HVEM or nectin-1) fused to scFv targeting tumor antigens.

In one pioneering study by Joseph C. Glorioso et al, a soluble adapter (P–V528LH) was developed by fusing the nectin-1 V domain with an EGFR-specific scFv. This adapter enabled HSV to enter EGFR-expressing CHO cells nearly as efficiently as via cell surface nectin-1. However, infection through nectin-1 remained active and depended significantly on cell-surface glycosaminoglycans.^144^ Subsequently, a bispecific adapter (scCEA-HveA) was engineered by fusing an scFv specific to the carcinoembryonic antigen (CEA) with HVEM's gD-binding domain. This adapter facilitated the infection of ectopic CEA-expressing CHO–K1 cells and CEA-positive gastric cancer cells (MKN45) by a nectin-1-detargeted mutant virus (K-222/3NI). The detargeted gD (R222N/F223I) retained the ability to bind to functional HVEM, which is predominantly expressed in immune cells, reducing off-target effects. Notably, this modification also allowed limited infection of nectin-1-positive cells, enabling adapter-independent viral spread to some extent. *In vitro* studies confirmed efficient cell-to-cell viral spread after adapter-mediated infection, while *in vivo* studies demonstrated reduced tumor growth, suggesting that this approach offers a promising strategy for tumor-specific oHSV-1 delivery.^145^ Interestingly, adapter-mediated infection relied on low pH-dependent endocytosis, similar to wild-type HSV-1 infection of nectin-1-expressing CHO cells, indicating that the adapter does not significantly alter the viral entry mechanism.^146^

While soluble adapter-based retargeting is effective, its reliance on purified proteins and repeated administration poses practical challenges. To address this, Hyun-Yoo Joo et al developed a dual-targeting oHSV platform by incorporating two distinct retargeting modalities into a nectin-1-detargeted strain (KOSG, gD: R222N/F223I). In this system, modified gH proteins with scFv targeting EpCAM or HER2 were used to initiate infection selectively in tumor cells. Subsequently, the virus encoded an adapter consisting of an anti-EpCAM scFv fused to the gD-binding N-terminal region of HVEM. This secreted adapter facilitated the lateral spread of progeny virus through a gD-dependent entry pathway.^147^ This dual-targeting strategy eliminated the need for exogenously added adapters, enhancing specific lateral spread between target cells via self-produced adapter molecules. EA-EgH-D, the resulting virus, exhibited superior entry and spread compared with both gH-modified and wild-type strains *in vitro*. In a subcutaneous xenograft model, a single intravenous injection of the EpCAM-specific virus eradicated detectable tumors without causing harm to immunocompetent mice, even at equivalent dosages. These results demonstrate the potential of adapter-based dual-targeting oHSV platforms as highly effective, versatile, and systemically administered anti-cancer therapies.

### Systemic delivery of oncolytic HSV

Currently, oHSV-1 is primarily administered via intratumoral injection, targeting accessible tumors. However, given that metastasis is the leading cause of cancer-related deaths, systemic delivery of oHSV-1 offers the potential to reach distant micrometastases and stimulate a robust anti-tumor immune response. Preclinical studies have demonstrated the efficacy of intravenously administered oHSV-1, such as HSV1716,^148^ Ld0-GFP,^149^ and MVR-T3011,^150^ in mouse models of liver and brain tumors. Phase I clinical trials of Seprehvir (HSV1716) in patients with extracranial solid tumors revealed good tolerability and no neurotoxicity.^151^ Additionally, hepatic artery infusion of NV1020 minimized toxicity, restricted liver metastases, and prolonged survival in metastatic colorectal cancer patients during a phase I/II trial.^152^

To address off-target infections, particularly in immunocompromised patients, viral specificity can be enhanced through retargeting strategies. For instance, intravenous delivery of EGFR-targeted oHSV1 (gD:SD2) significantly inhibited tumor growth in a mouse model of glioblastoma multiforme.^131^ Retargeted strains, such as R337 (Her2) and R405 (hPSMA), selectively infected tumor-bearing lung tissues and subcutaneous tumors, showing enhanced efficacy when combined with immune checkpoint inhibitors.^153^ Of note, deletion of gC amino acids critical for glycosaminoglycan interaction further reduced off-target infections in strains such as R337.^154^ In addition, fusion of the CD47 ectodomain with gC enabled oHSV1 to evade clearance by the mononuclear phagocyte system during systemic delivery, thereby improving delivery efficiency and prolonging viral persistence in tumors.^155^

However, despite the existing evidence that supports the safety of systemic administration of oHSV-1, its effectiveness continues to encounter challenges. For instance, the presence of a widespread pre-existing antiviral immunity results in rapid clearance of the oncolytic virus through neutralizing antibodies, which significantly reduces the viral load reaching tumor sites.^153^^,^^156^ Additionally, human lung, liver, and spleen reticuloendothelial tissue contains a significant number of macrophages that possess strong clearance capabilities for OVs.^157^

To avoid the dilution effect in the blood or clearance by the liver mononuclear phagocyte system, several strategies, such as cell-based carriers or nanotechnology approaches, are proposed to deliver viruses to tumors. Mesenchymal stem cells are promising carriers due to their tumor tropism under hypoxic and inflammatory conditions.^158^ Studies have shown that arming mesenchymal stem cells with different strains of oHSV-1 (*e.g.*, G47Δstrain or Her2 retargeted R-LM249 strain) could successfully track metastasis and prolong the survival of mice bearing brain metastatic melanomas^159^ or glioblastoma multiforme,^160^ or lung and brain metastases.^161^ In addition, nanoengineering is also used to modify OVs to enhance the serum stability and tumor targeting. Fu et al first proved that liposome-encapsulated viral genomic DNA, capsid, or intact virion could transfect target cells *in vitro* and *in vivo* and produce infectious virus.^162^ More importantly, the liposome/DNA could evade the host neutralizing antibodies during systemic administration. Another, the systemic administration of HSV 1716 co-assembling with magnetic nanoparticles via electrostatic interactions could reach the tumor site with magnetic guidance.^163^ These nanomagnets protect viruses from neutralization by antibodies in the body, thereby significantly augmenting their anti-tumor effects in a syngeneic mouse model of breast cancer.^163^ Furthermore, combining oHSV-1 with a copper-chelating agent ATN-224 improved serum stability and enhanced anti-tumor effects by inhibiting angiogenesis.^164^

## Conclusion and perspectives

Over the past three decades, remarkable progress has been made in the field of tumor-specific engineering of oHSV-1. These achievements stem largely from a comprehensive understanding of the roles of viral glycoproteins such as gD, gC, gB, and gH/gL in mediating viral attachment and entry into host cells. Structural and functional analyses, including crystal structures of these glycoproteins interacting with receptors like nectin-1 and HVEM, have provided the foundation for designing retargeted viruses that selectively target tumor-associated antigens without attenuating oncolytic activity. These advancements, along with tumor-specific markers and monoclonal antibodies, have enabled the development of highly specific and potent oHSV constructs.

Despite these advances, challenges remain, particularly with off-target effects and alternative entry pathways. While retargeting strategies have successfully detargeted HSV from receptors like nectin-1 and HVEM, alternative mechanisms of entry via glycoproteins such as gB and gH complicate systemic delivery, especially in the bloodstream. To address these challenges, synergistic modifications across multiple glycoproteins may mimic natural viral glycoprotein interactions. Such strategies could enhance cell entry efficiency and reduce off-target effects, making systemic delivery safer and more effective. Additionally, syncytial mutations associated with gB hyperfusogenic activity hold significant potential for improving intra-tumoral spread. By promoting rapid cell-to-cell infection and reducing reliance on extracellular virus dissemination, these mutations could help bypass neutralizing antibodies and increase therapeutic efficacy.

Advancements in gene editing technologies, such as bacterial artificial chromosomes, CRISPR-Cas9, and emerging methods like LSR recombination, are further accelerating the pace of oHSV engineering.^156^ These tools allow for precise, scalable genetic modifications and the development of next-generation oHSV strains tailored for specific applications. Integrating these technologies with immunomodulatory strategies, such as arming oHSVs with cytokines or immune checkpoint inhibitors, can amplify anti-tumor immune responses while overcoming immunosuppressive tumor environments. Combining oHSV therapy with immune-based approaches represents a promising avenue to maximize therapeutic efficacy.

**Figure 4:**
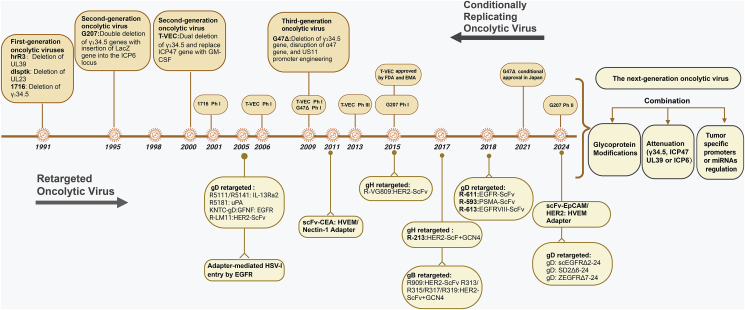
Historical timeline of milestones in the development of oncolytic virotherapy.

Another promising direction for future oHSV-1 development lies in the integration of dual-layered regulation strategies that combine glycoprotein-based retargeting with tumor-specific replication control mechanisms. By simultaneously modifying viral entry glycoproteins (*e.g.*, gD, gH, or gB) to restrict infection to tumor-associated receptors, and regulating viral gene expression through tumor-selective promoters (*e.g.*, nestin, HIF-1α), miRNA response elements (*e.g.*, miR-124 targeting), or classical attenuation deletions (*e.g.*, γ34.5 or UL39), it is possible to achieve precise control over both infection and replication. Such dual strategies ensure that even if the virus enters off-target tissues, it cannot replicate efficiently. This layered safety architecture represents a critical step toward enhancing the clinical safety and specificity of systemically delivered oHSV-based therapies, especially for metastatic and neurologically sensitive tumors (Fig. 4).

Structural biology will continue to play a pivotal role in the design of next-generation oHSV strains, providing detailed insights into glycoprotein-receptor interactions and guiding the development of fully retargeted viruses. These efforts aim to create highly specific, safe, and effective oncolytic viruses capable of addressing the challenges of metastatic and inaccessible tumors. The application of such precision engineering will bridge the gap between experimental therapeutics and clinical translation, bringing transformative cancer treatments closer to reality.

As the field moves forward, multidisciplinary collaboration between structural biologists, immunologists, and virologists will be critical to overcoming remaining obstacles. Integrating innovative glycoprotein engineering, advanced gene editing, and immunotherapy will unlock the full potential of oHSV as a powerful tool in fighting against cancer. With sustained research and development, oHSV-based therapies hold the promise of becoming a cornerstone of modern oncology, offering new hope to patients with metastatic and refractory cancers.

## CRediT authorship contribution statement

**Yufang Zou:** Writing – original draft, Conceptualization. **Juan Tao:** Writing – original draft. **Yingzheng Gao:** Writing – original draft, Visualization. **Jixuan Wang:** Writing – original draft. **Pengfei Wang:** Writing – original draft, Visualization. **Jingyuan Yan:** Writing – review & editing. **Zuqing Nie:** Writing – original draft. **Dewei Jiang:** Writing – review & editing, Conceptualization. **Xinwei Huang:** Writing – review & editing, Visualization, Funding acquisition, Conceptualization.

## Funding

This work was partly supported by grants from the 10.13039/501100001809National Natural Science Foundation of China (No. 82260486, 82173014), Yunnan Revitalization Talent Support Program (China) (Young Shcolar to X.H. and D.J.), Yunnan Medical Discipline Leader Training Project (China) (No. D-2024031 to X.H.), and Graduate Innovation Fund of Kunming Medical University, Yunnan, China (No. 2024S086), and Biomedical Projects of Yunnan Key Science and Technology Program (China) (No. 202302AA310046 to D.J.).

## Conflict of interests

The authors declared no conflict of interests.
